# A large-scale database of T-cell receptor beta (TCRβ) sequences and binding associations from natural and synthetic exposure to SARS-CoV-2

**DOI:** 10.21203/rs.3.rs-51964/v1

**Published:** 2020-08-04

**Authors:** Sean Nolan, Marissa Vignali, Mark Klinger, Jennifer N. Dines, Ian M. Kaplan, Emily Svejnoha, Tracy Craft, Katie Boland, Mitch Pesesky, Rachel M. Gittelman, Thomas M. Snyder, Christopher J. Gooley, Simona Semprini, Claudio Cerchione, Massimiliano Mazza, Ottavia M. Delmonte, Kerry Dobbs, Gonzalo Carreño-Tarragona, Santiago Barrio, Vittorio Sambri, Giovanni Martinelli, Jason D. Goldman, James R. Heath, Luigi D. Notarangelo, Jonathan M. Carlson, Joaquin Martinez-Lopez, Harlan S. Robins

**Affiliations:** Adaptive Biotechnologies; Adaptive Biotechnologies; Adaptive Biotechnologies; Adaptive Biotechnologies; Adaptive Biotechnologies; Adaptive Biotechnologies; Adaptive Biotechnologies; Adaptive Biotechnologies; Adaptive Biotechnologies; Adaptive Biotechnologies; Adaptive Biotechnologies; Microsoft Research; University of Bologna; Istituto Scientifico Romagnolo per lo Studio e la Cura dei Tumori; Istituto Scientifico Romagnolo per lo Studio e la Cura dei Tumori; National Institute of Allergy and Infectious Diseases, National Institutes of Health; National Institute of Allergy and Infectious Diseases, National Institutes of Health; Complutense University; Complutense University; University of Bologna; Istituto Scientifico Romagnolo per lo Studio e la Cura dei Tumori; Swedish Medical Center, Seattle, WA, USA and Division of Allergy and Infectious Diseases, University of Washington, Seattle, WA, USA; Institute for Systems Biology; National Institute of Allergy and Infectious Diseases, National Institutes of Health; Microsoft Research; Complutense University; Adaptive Biotechnologies

**Keywords:** ImmuneCODE, TCR, SARS-CoV-2, immunosequencing

## Abstract

We describe the establishment and current content of the ImmuneCODE™ database, which includes hundreds of millions of T-cell Receptor (TCR) sequences from over 1,400 subjects exposed to or infected with the SARS-CoV-2 virus, as well as over 135,000 high-confidence SARS-CoV-2-specific TCRs. This database is made freely available, and the data contained in it can be downloaded and analyzed online or offline to assist with the global efforts to understand the immune response to the SARS-CoV-2 virus and develop new interventions.

## Introduction

The emergence of SARS-CoV-2 in December of 2019^1^ and the ensuing pandemic declared by the WHO at the end of January 2020^2^ created an urgent need to understand the disease and its causative agent. Initial studies have shown a strong T-cell based adaptive immune response^3^,^4^,^5^, but its detailed nature remains uncharacterized. We therefore applied our previously described immunoSEQ® Assay^6^,^7^,^8^ and MIRA™ tool^9^,^10^ to deepen the understanding of the adaptive immune response to SARS-CoV-2 infection in support of COVID-19 research.

To generate these data, we partnered with Microsoft, Illumina, Labcorp/Covance, and health organizations across the world to generate the ImmuneCODE database described herein. These data are being made freely available to the scientific community so that any researcher, public health official or organization can utilize the data to accelerate ongoing global efforts to develop better diagnostics, vaccines and therapeutics, as well as to answer important questions about the virus.

The database consists of two distinct but related datasets. (A) **The immunoSEQ dataset** includes 1,414 deeply- sampled TCRb repertoires from subjects who at the time of sampling either had been exposed to, were actively suffering from, or had recovered from COVID-19. These data originate from two sources (Table 1): ImmuneRACE (Immune Response Action to COVID-19 Events), an ongoing prospective study enrolling participants across the U.S. to decode how immune systems detect and respond to the virus, which includes self-reported demographic and clinical data, and (2) thousands of de-identified geographically and ethnically diverse patient blood samples collected by institutions around the world. (B) **The MIRA dataset** maps TCRs binding to SARS-Cov-2 virus epitopes, and includes data obtained from exposed subjects and naïve controls. In total, the MIRA dataset includes more than 135,000 high-confidence SARS-CoV-2-specific TCRs.

The data include varying degrees of demographic and clinical information (as allowed by each institution and corresponding IRB). Additional metadata may be added in the future.

The ImmuneCODE database will continue to grow both as we continue to recruit participants to ImmuneRACE and as we add samples collected by additional institutions. This will result in additional T-cell repertoires of exposed and infected individuals and SARS-CoV-2-specific TCRs, allowing the association of T-cell signatures with disease and outcomes. We hope that this freely available resource will inform our understanding of the immune response to the virus and that it will be useful for researchers around the world by accelerating their work in basic and applied immunology, thus contributing to the development of new therapeutic and preventive measures.

## Results

### Dataset Access

**Figure 1:**
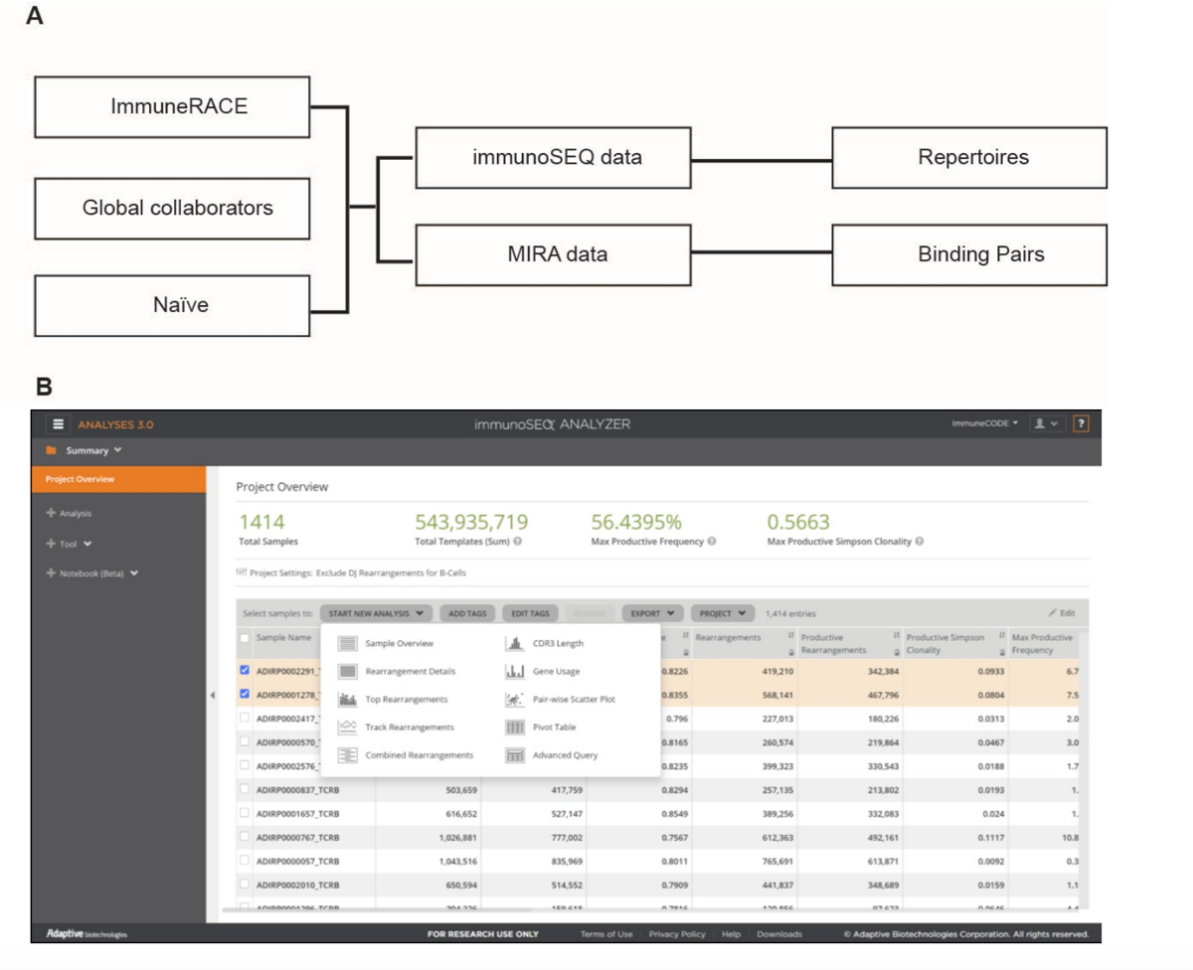
The ImmunoCODE Database (A) Schema of the included data (B) Screenshot of the landing page.

The ImmuneCODE database includes both immunoSEQ and MIRA data (Figure 1 a). and is being shared through the immuneACCESS® data portal (Figure 1 b), which enables the export of complete or selected data, as well as real-time analysis using a rich suite of custom-built tools. Data are available at (https://clients.adaptivebiotech.com/pub/covid-2020;_DOI 10.21417/ADPT2020COVID). Note that the dataset will continue to grow over time; subjects described in this article can be identified by selecting samples with the “ImmunoCODERelease” tag value “002”.

### immunoSEQ data

The ongoing immueRACE study aims to enroll 1,000 subjects who have been exposed to, are currently infected with, or have recovered from COVID-19. The current release of the database includes T-cell repertoire data from the first 160 participants in the study (including multiple samples from some subjects): new data will be added as it is generated. This release also includes T-cell repertoire data from 1,254 subjects from 6 global collaborators (Table 1): new T-cell repertoires may be generated both by adding new samples from these ongoing studies, and by incorporating additional institutions to this effort.

These data were generated from participant samples using the TCRb immunoSEQ Assay as previously described6,7,8. They include a list of unique TCRb rearrangements found in each analyzed sample, a count for each rearrangement, and sample-level metadata. Certain pre-configured analyses we believe will be most used will also be available through immuneACCESS, so that users do not need to recreate them. The data can be exported using dedicated links on the immuneACCESS project page for offline analysis.

By default the immunoSEQ Analyzer includes many metadata fields that are useful across different research contexts; Tables 2 and 3 describe the key fields most relevant to this dataset and should be useful to users interested in understanding the definitions of the different fields. Specifically, Table 2 describes the sample level fields included, whereas Table 3 describes the sequence-level fields. The amount of metadata available varies by source and participant; we include all available, uncurated metadata for each sample in the “sample_tags” field. In almost all cases, these include de-identified subject IDs, COVID-19 status, age in years, and sex.

### MIRA data

Antigen-specific TCRs were identified using the ‘Multiplex Identification of Antigen-Specific T-Cell Receptors Assay (MIRA)^9^,^10^. MIRA is a high-throughput multiplex tool, enabling the identification of antigen-specific TCR to large numbers of query antigens (hundreds to thousands at a time and in parallel) by combining immune assays with T-cell receptor sequencing. We use cell sorting based on the upregulation of activation markers to separate a population of antigen-specific T cells. This positive population is sequenced via immunoSEQ, and clonotypes specific to antigen are identified by virtue of enrichment in the positive population compared to a sample of unenriched or unsorted T cells.

With the goal of identifying SARS-CoV-2-specific TCRs, we interrogated T-cell repertoires from both healthy donors and COVID-19 patients. Input cell types used varied and included PBMCs from healthy donors or COVID- 19 patients, and naïve T cells from healthy donors. To maximize TCR yield per experiment, we expanded T cells from both types of input cells. When starting with PBMCs from either healthy donors or COVID-19 patients, T cells were expanded polyclonally with soluble anti-CD3. When starting with naïve CD8+ T cells from healthy donors, T cells were expanded following co-culture with monocyte-derived DCs loaded with a pool of all peptides derived from SARS-CoV-2.

We used two different MIRA tool approaches: peptide- or transgene-based. Both enable the identification of antigen-specific TCRs, however the transgene-based approach enables identification of TCRs that are specific to epitopes encoded and presented by APCs following expression upon transfection of transgenes. This approach enables us to distinguish the subset of TCRs that respond to endogenously-presented epitopes rather than those that only respond to exogenously loaded peptides. Binding or activation following a multimer stain or incubation with peptides is therefore not an indicator of whether a T cell is specific to an endogenously presented epitope. The underlying assumption for any immunological assay involving multimers or exogenously loaded peptides is that the epitope being tested is actually a presented epitope. For well-characterized epitopes this assumption is reasonable, however when querying large numbers of novel epitopes from a novel virus (SARS-CoV-2, for example) the risk for false positives (defined as TCRs specific to a never-before tested peptide that was exogenously loaded), is higher.

In total, the MIRA dataset includes more than 135,000 high-confidence high-confidence SARS-CoV-2-specific TCRs. These data are made available as a set of downloadable files “ImmuneCODE MIRA Release 002.zip”, which can also be accessed through the immuneACCESS project page.

The dataset includes experiments from three MIRA panels. Two of these panels, named “minigene_Set1” and “minigene_Set2”, targeted large protein sequences intended to narrow down which parts of the genome generally elicit immune response. The third panel, named “C19_cI”, targeted individual peptides or small groups of peptides. Most of the MIRA data included in this dataset corresponds to the C19_cI panel.

Tables 4 through 9 describe the MIRA data included in the database, as follows: Table 4 (subject-metadata.csv) includes available metadata for each sample from subjects included in the MIRA experiments (both in the two minigene and in the peptide panels described above). HLA types are provided when available. Missing values are generally represented with “N/A”, except for HLA types, where missing data is represented as an empty string. Note that the metadata contained in this file relates to the MIRA results, and is distinct from the immunoSEQ-related metadata (I.e. “tags” in the tables above). Table 5 (orfs.csv) includes the genomic location of the MIRA targets as per GenBankl 1. Table 6 (minigene-hits.csv) contains counts of the number of unique TCRs that bound to targets within the “minigene_Set1” and “minigene_Set2” MIRA panels, while Table 7 (minigene- detail.csv) describes the identity of the TCRs bound per target for both minigene MIRA panels. Finally, Table 8 (peptide-hits.csv) contains counts of the number of unique TCRs that bound to targets within the “C19_cI” MIRA panel, while Table 9 (peptide-detail.csv) describes the identity of the TCRs bound per target for the C19_cl MIRA panel.

## Discussion

To assist in the understanding of the adaptive immune response to SARS-CoV-2, we generated the freely-available ImmuneCODE database described herein, which includes a dataset of TCR rearrangements observed in individuals exposed to, infected with or recovered from COVID-19, and describes the ability of a subset of these TCRs to recognize SARS-CoV-2 epitopes. These data are provided to the scientific community at large with the goal of contributing to their research efforts to develop novel interventions to prevent and treat COVID-19 infections.

In-depth understanding of the T-cell response to the COVID-19 causative agent may improve the accuracy of existing testing paradigms, and potentially provide an assessment of immunity. These immune response data may help to solve two of the key challenges we are facing in the current diagnostic paradigm, namely (1) detection of the virus in infected people who are asymptomatic, and (2) detection of past infections later than serology and in other cases where antibodies are not present.

Additionally, it is possible that identifying and tracking the T-cell response to the virus may provide insight as to the severity of a patient’s illness, the length of any post-infection immunity period, the effect of the infection on individuals with cancer and other conditions conferring higher risk of severity, and the potential efficacy of vaccines in development.

## Online Methods

### ImmuneRACE experimental cohort and study approval

The ImmuneRACE study is a prospective, single group, multi-cohort, exploratory study of unselected eligible participants exposed to, infected with, or recovering from COVID-19 (NCT04494893). Participants, aged 18 to 89 years and residing in 24 different geographic areas across the United States, were consented and enrolled via a virtual study design. Cohorting was based on participant-reported clinical history following the completion of both a screening survey and study questionnaire.

Cohort 1 included participants exposed within 2 weeks of study entry to someone with a confirmed COVID-19 diagnosis, either based on positive PCR testing or clinician diagnosis. Cohort 2 participants included those clinically diagnosed by a physician or with positive laboratory confirmation of active SARS-CoV-2 infection via PCR testing. Cohort 3 included participants previously diagnosed with COVID-19 disease who have been deemed recovered based on two consecutive negative nasopharyngeal or oropharyngeal (NP/OP) PCR tests, clearance by a healthcare professional, or the resolution of symptoms related to their initial COVID-19 diagnosis. The ImmuneRACE study was approved by Western Institutional Review Board (WIRB reference number 1–1281891- 1, Protocol ADAP-006). All participants were consented for sample collection and metadata use via electronic informed consent processes.

Both whole blood and serum and a nasopharyngeal or oropharyngeal swab were collected from participants by trained mobile phlebotomists. Blood samples were shipped frozen or at room temperature to Adaptive Biotechnologies for processing, including, but not limited to, DNA extraction, and TCRb analysis via the immunoSEQ Assay (Adaptive Biotechnologies, Seattle, WA) from DNA extracted from blood samples (Table 1). NP/OP swabs and serum were sent to Covance/Labcorp for further testing. An electronic questionnaire was administered to collect information pertaining to the participant’s medical history, symptoms, and diagnostic tests performed for COVID-19 disease. Participants have the option to undergo additional blood draws and questionnaires over 2 months.

### Global data collaborations

Whole blood samples were collected in K2EDTA tubes based on each institution’s protocol and supervised by their respective Institutional Review Board. Samples were stored at the institution and sent to Adaptive as frozen whole blood, isolated PBMC or DNA extracted from either sample type for TCRb analysis via the immunoSEQ

Assay (see Table 1). Samples provided by the NIAID were collected under approval by Comitato Etico Provinciale (protocol NP-4000), by Comitato Etico, Ospedale San Gerardo Monza (protocol COVID-STORM) and by Comitato Etico Pavia Fondazione IRCCS Policlinico San Matteo, Pavia (protocol 20200037677). Whole blood samples from DLS (Discovery Life Sciences, Huntsville, AL) were collected under Protocol DLS13 for collection of remnant clinical samples. From Bloodworks Northwest (Seattle, WA), volunteer donors recovered from COVID-19 were consented and collected under the Bloodworks Research Donor Collection Protocol BT001. Samples were processed for PBMC and donor data reported by the Biological Products division of Bloodworks NW under standard operating procedures.

### Sample analysis

A subset of the samples were processed for both T-cell receptor variable beta chain sequencing and MIRA, and another subset was processed only by one of these approaches. For each subject included in the dataset, SubjectID can be used to determine which assay the samples were processed in.

### T-cell receptor variable beta chain sequencing

Immunosequencing of the CDR3 regions of human TCRβ chains was performed using the immunoSEQ Assay as previously described6,7,8. In brief, extracted genomic DNA was amplified in a bias-controlled multiplex PCR, followed by high-throughput sequencing. Sequences were collapsed and filtered in order to identify and quantitate the absolute abundance of each unique TCRβ CDR3 region for further analysis.

### Multiplexed Identification of TCR Antigen Specificity (MIRA)

To identify antigen-specific TCRs, T cells derived post-expansion from either of the above input cell types were used for the MIRA tool. Antigen-specific TCRs were identified as previously described9,10. Briefly, T cells were incubated overnight with MIRA peptide pools, and the antigen-specific subset was identified by CD137 upregulation. Following addition of peptides, cells were incubated at 37°C for ~18 hours. At the end of the incubation, replicate wells of cells were harvested from the culture and pooled and then stained with antibodies for analysis and sorting by flow cytometry. Cells were then washed and suspended in PBS containing FBS (2%), 1mM EDTA and 4,6-diamidino- 2-phenylindole (DAPI) for exclusion of non-viable cells. Cells were acquired and sorted using a FACS Aria (BD Biosciences) instrument. Sorted antigen-specific (CD3+CD8+CD137+) T cells were pelleted and lysed in RLT Plus buffer for nucleic acid isolation. Analysis of flow cytometry data files was performed using FlowJo (Ashland, OR).

RNA was isolated using AllPrep DNA/RNA mini and/or micro kits, according to manufacturer’s instructions (Qiagen). RNA was reverse transcribed to cDNA using Vilo kits (Life Technologies). TCRβ amplification, sequencing and clonotype determination were performed as described in the ‘T-cell receptor variable beta chain sequencing’section above.

### MIRA tool design

T-cell populations were exposed to pooled peptides or transgenes in a combinatoric format, similar to the approach described in reference 10. According to the MIRA panel design, each antigen is strategically placed in a subset of K unique pools while being omitted from the remaining pools (total pools = N). This design allows for antigens to be placed into a unique combination of N choose K occupancies (or also referred to as “addresses”), and allows for increased economies of scale as the number of replicate pools (N) increases. In order to estimate an empirical false discovery rate and gauge assay quality, we purposefully left > 40% of the unique occupancies empty to assess the rate at which are clones are spuriously sorted and detected in K pools with no query antigen present (hereinafter referred to as invalid TCR associations).

### Matching clonotypes to antigens

T cells were aliquoted into 11 pools, and activated T cells were sorted using T-cell markers after overnight stimulation, as described previously^10^. These putative antigen responding cells were set aside to characterize the T-cell clonotypes present in each sorted pool using the immunoSEQ Assay as described above. After immunosequencing, we examined the behavior of T-cell clonotypes by tracking the read counts of each unique TCRb sequence across each sorted pool. True antigen-specific clones should be specifically enriched in a unique occupancy pattern that corresponds to the presence of one of the query antigens in K pools. We have reported on methods to assign antigen specificity to TCR clonotypes previously^12^; in addition we also developed a non-parametric Bayesian model to compute the posterior probability that a given clonotype is antigen specific. This model uses the available read counts of TCRs to estimate a mean-variance relationship within a given experiment and as well as the probability that a clone will have zero read counts due to incomplete sampling of low frequency clones. Together, this model takes the observed read counts of a clonotype across all N pools and estimates the posterior probability of a clone responding to all possible N choose K addresses and an additional hypothesis that a clone is activated in all pools (truly activated, but no specific to any of our query antigens). To define antigen specific clones, we identified TCR clonotypes assigned to a query antigen from this model with a posterior probability >= 0.9.

## Figures and Tables

**Table 1:** List of available samples per dataset, including number of samples, institution and description of sample type and source.

Name of the dataset	Sample Count	Institution	Study Description
COVID-19-Adaptive	160	Adaptive	ImmuneRACE and Antigen Map COVID19: immune response to COVID-19 (with Microsoft); cDNA or gDNA from T cells, B-cell depleted T cells, or PBMCs
COVID-19-BWNW	50	Bloodworks Northwest	Whole blood samples from convalescent patients collected at Bloodworks Northwest (Seattle, WA)
COVID-19-DLS	433	Discovery Life Sciences	Whole blood samples collected during routine patient care in acute and convalescent phases procured through Discovery Life Sciences (Huntsville, AL)
COVID-19-ISB	157	Institute for Systems Biology	Whole blood samples collected under the INCOVE project at Providence St. Joseph Health (Seattle, WA). Patients were enrolled during the active phase and monitored through disease
COVID-19-NIH/NIAID	357	National Institute for Allergy and Infectious Diseases (NIAID)	Whole blood samples were collected in Brescia, Monza and Pavia (Italy) during active infection, and provided to the NIAID (Bethesda, MD) for DNA extraction
COVID-19-HUniv12Oct	193	Hospital Universitario 12 de Octubre	Whole blood samples were collected at the Hospital Univesitario 12 de Octubre (Madrid, Spain) during the active or convalescent phase
COVID-19-IRST/AUSL	64	Istituto Scientifico Romagnolo per lo Studio e la Cura dei Tumori (IRST) / AUSL-Romagna	Whole blood samples were collected by IRST/AUSL (Romagna, Italy) during active infection

**Table 2:** Sample-level fields included in the immunoSEQ Analyzer for immunoSEQ data.

Field name in immunoSEQ Analyzer	Field name in Advanced Query / Packaged data	Type	Description
Sample Name	sample_name	string	Sample name. Values used are not meaningful and should be interpreted as an opaque identifier.
Nucleated Cells	sample_cells	integer	The total number of nucleated cells (includes immune and other cells). This value is based on the amplification of reference gene primers in the assay.
Total T Cells	total_t_cells	double	The total number of T cells in a sample as detected by the assay.

**Table 3:** Sequence-level fields included in the immunoSEQ Analyzer for immunoSEQ data.

Total Templates (Sum)	total_templates	integer	The sum of template counts for all productive and unproductive rearrangements in the sample. Expect this value to be slightly larger than Total Templates (Aggregate) found in immunoSEQ Analyzer due to rounding of template counts for individual rearrangements.
Total Productive Templates (Sum)	productive_templates	integer	The sum of template counts for all productive rearrangements in the sample. Expect this value to be different than Total T cells, due to rounding of template counts for individual rearrangements.
Rearrangements	total_rearrangements	integer	The count of unique rearrangements identified in the sample. Each rearrangement may be present in multiple cells. A rearrangement is a particular nucleotide sequence generated through V(D)J recombination.
Productive Rearrangements	productive_rearrangements	integer	The count of unique rearrangements in the sample that are in-frame and do not contain a stop codon. Productive rearrangements can produce a functional protein receptor.
Productive Simpson Clonality	productive_simpson_clonality	double	Productive Simpson Clonality is calculated for a sample as the square root of Simpson’s diversity index for all productive rearrangements. Values for clonality range from 0 to 1. Values near 1 represent samples with one or a few predominant rearrangements (monoclonal or oligoclonal samples) dominating the observed repertoire. Clonality values near 0 represent more polyclonal samples.
Fraction T Cells of Nucleated Cells	fraction_productive_of_cells	fraction (0.0 – 1.0)	The fraction of T cells within the total nucleated cell count (T cells and non-T cells). This value is calculated by dividing the number of Productive Templates by Nucleated Cells.
Max Productive Frequency	max_productive_frequency	fraction (0.0 – 1.0)	The maximum Productive Frequency value found within a sample. Productive Frequency for a specific productive rearrangement is calculated as the Templates for a specific rearrangement divided by the Sum of Productive Templates for a sample.
HLA Class I	hla_class_i	csv	The HLA Class I type metadata provided by the customer upon receipt of specimen; usually presented as a comma-delimited string of Class I alleles, e.g. “A*02:01,A*23:01,B*40:02...”
HLA Class II	hla_class_ii	csv	The HLA Class II type metadata provided by the customer upon receipt of specimen; usually presented as a comma-delimited string of Class II alleles, e.g. “DPA1*02:02,DPA1*02:02,DPB1*...”
Sample Tags	sample_tags	csv	The metadata tags associated with this sample. Sample tags are either from the Adaptive Tag Library, or project-specific tags defined and created by the sample owner*

**Table 4:** Metadata for Subjects included in the MIRA data

Field name in immunoSEQ Analyzer	Field name in Advanced Query / Packaged data	Data type	Description
Rearrangement	rearrangement	string	A particular nucleotide sequence generated through V(D)J recombination, as detected by the immunoSEQ assay.
Extended Rearrangement	extended_rearrangement	string	The full length TCR imputed via algorithm for the Rearrangement; includes the full CDR1, CDR2 and CDR3 region.
Bioidentity	bio_identity	string	T cell bioidentity refers to the overall protein sequence of a T cell receptor. This is defined as the identified V gene, the amino acid sequence of the CDR3 region and the identified J gene. Two rearrangements with the same bioidentity would be expected to demonstrate the same binding and activation behavior. Data Format: [Amino Acid]+[v_gene]+[j_gene]
Amino Acid	amino_acid	string	The amino acid translation of the unique nucleotide rearrangement in the identified CDR3 region. Only productive rearrangements can be translated. Productive rearrangements are in-frame, do not contain a stop codon and can produce a functional protein receptor.
Templates	templates	integer	The total number of templates for a specific rearrangement in the sample.
Frame Type	frame_type	enum (In, Out, Stop)	The functional state of a rearrangement: in-frame (productive), out-of-frame, or containing a stop codon.
Rearrangement Type	rearrangement_type	string	The type of rearrangement process that generated a specific rearrangement.
Productive Frequency	productive_frequency	fraction (0.0 – 1.0)	The frequency of a specific productive rearrangement among all Productive Rearrangements within a sample. Calculated as the Templates for a specific rearrangement divided by the Sum of Productive Templates for a sample.
CDR1 Index	cdr1_start_index	integer	The index into the Extended Rearrangement string at which the CDR1 region begins.
CDR1 Rearrangement Length	cdr1_rearrangement_length	integer	The length (in characters) of the CDR1 region within Extended Rearrangement.
CDR2 Index	cdr2_start_index	integer	The index into the Extended Rearrangement string at which the CDR2 region begins.
CDR2 Rearrangement Length	cdr2_rearrangement_length	integer	The length (in characters) of the CDR2 region within Extended Rearrangement.
CDR3 Index	cdr3_start_index	integer	The index into the Extended Rearrangement string at which the CDR3 region begins.
CDR3 Length	cdr3_length	integer	The length of the CDR3 in nucleotides, starting from the first base of the codon for the conserved cysteine in the V gene through the last base of the codon for the conserved residue in the J gene that ends the CDR3.
V Index	v_index	integer	The index within the full nucleotide sequence that denotes the Cysteine beginning the CDR3.
N1 Index	n1_index	integer	The index within the full nucleotide sequence that denotes the start of the N1 (VD) region.
D Index	d_index	integer	The index within the full nucleotide sequence that denotes the start of the D region.
N2 Index	n2_index	integer	The index within the full nucleotide sequence that denotes the start of the N2 (DJ) region.
J Index	j_index	integer	The index within the full nucleotide sequence that denotes the start of the J region.
V Deletions	v_deletions	integer	The number of nucleotides deleted from the V gene during recombination.
N1 Insertions	n2_insertions	integer	The number of nucleotides inserted in the N1 (VD) junction during recombination.
D3 Deletions	d3_deletions	integer	The number of nucleotides deleted from the 3’ end of the D gene during recombination.
D5 Deletions	d5_deletions	integer	The number of nucleotides deleted from the 5’ end of the D gene during recombination.
N2 Insertions	n1_insertions	integer	The number of nucleotides inserted in the N2 (DJ) junction during recombination.
J Deletions	j_deletions	integer	The number of nucleotides deleted from the J gene during recombination.
Chosen J Allele	chosen_j_allele	string	The j-gene allele that was used to impute Extended Rearrangement.
Chosen J Family	chosen_j_family	string	The j-gene family that was used to impute Extended Rearrangement.
Chosen J Gene	chosen_j_gene	string	The j-gene that was used to impute Extended Rearrangement.
Chosen V Allele	chosen_v_allele	string	The v-gene allele that was used to impute Extended Rearrangement.
Chosen V Family	chosen_v_family	string	The v-gene family that was used to impute Extended Rearrangement.
Chosen V Gene	chosen_v_gene	string	The v-gene that was used to impute Extended Rearrangement.
D Allele	d_allele	string	The identified D Gene Allele that contributed to a specific rearrangement.
D Allele Ties	d_allele_ties	csv	A comma-separated list of equivalently- scored D Gene Alleles identified during annotation.
D Family	d_family	string	The identified D Gene Family that contributed to a specific rearrangement.
D Family Ties	d_family_ties	csv	A comma-separated list of equivalently- scored D Gene Families identified during annotation.
D Gene	d_gene	string	The identified D Gene that contributed to a specific rearrangement.
D Gene Ties	d_gene_ties	csv	A comma-separated list of equivalently- scored D Genes identified during annotation.
D Resolved	d_resolved	string	A concise string identifying the most specific D Gene family, gene or allele identified during annotation.
J Allele	j_allele	string	The identified J Gene Allele that contributed to a specific rearrangement.
J Allele Ties	j_allele_ties	csv	A comma-separated list of equivalently- scored J Gene Alleles identified during annotation.
J Family	j_family	string	The identified J Gene Family that contributed to a specific rearrangement.
J Family Ties	j_family_ties	csv	A comma-separated list of equivalently- scored J Gene Families identified during annotation.
J Gene	j_gene	string	The identified J Gene that contributed to a specific rearrangement.
J Gene Ties	j_gene_ties	csv	A comma-separated list of equivalently- scored J Genes identified during annotation.
J Resolved	j_resolved	string	A concise string identifying the most specific J Gene family, gene or allele identified during annotation.
V Allele	v_allele	string	The identified V Gene Allele that contributed to a specific rearrangement.
V Allele Ties	v_allele_ties	csv	A comma-separated list of equivalently- scored V Gene Alleles identified during annotation.
V Family	v_family	string	The identified V Gene Family that contributed to a specific rearrangement.
V Family Ties	v_family_ties	csv	A comma-separated list of equivalently- scored V Gene Families identified during annotation.
V Gene	v_gene	string	The identified V Gene that contributed to a specific rearrangement.
V Gene Ties	v_gene_ties	csv	A comma-separated list of equivalently- scored V Genes identified during annotation.
V Resolved	v_resolved	string	A concise string identifying the most specific V Gene family, gene or allele identified during annotation.

**Table 5:** Genomic location of MIRA targets

Field	Value	Notes
Experiment	String	Opaque identifier for the MIRA experiment. This column joins to the *-details.csv files.
Subject	String	Opaque identifier for the subject (also the “sample” in the context of MIRA).
Cell Type	Enum	· PBMC · naive_CD8
Target Type	Enum	The MIRA panel for the experiment: · minigene_Set1 · minigene_Set2 · C19_cI (peptides)
Cohort	Enum	· Healthy (No known exposure) · COVID-19-Convalescent · COVID-19-Acute · COVID-19-Exposed
Age	Integer	In years or N/A
Gender	Enum	· M · F · N/A (other)
Race	String	Uncontrolled values or N/A
HLA	Multiple columns	HLA values as provided by the data source.

**Table 6:** Number of TCRs bound per target in the minigene MIRA panels

Field	Value	Notes
orf	String	The abbreviated name of an open reading frame. Joins to the “ORF”-related columns in *-hits.csv and *-details.csv files.
index_genome	Integer	The 1-based index of the first base of the ORF within the genome.
end_index_inclusive	Integer	The 1-based index of the last base of the ORF within the genome.

**Table 7:** Identity of TCRs bound per target in the minigene MIRA panels

Field	Value	Notes
ORF	String	The ORF in which this target is located.
ORF Genebank ID	String	The identifier for the sequence from which the target was selected.
Amino Acid	String	The protein sequence of the target.
Start Index in Genome	Integer	The 1-based index of the first base of the target within the genome.
End Index in Genome	Integer	The 1-based index of the last base of the target within the genome.
Hits	Integer	The unique count of TCRs identified as binding to the target, across all experiments.

**Table 8:** Number of TCRs bound per target in the peptide MIRA panel

Field	Value	Notes
TCR BioIdentity	String	Represents the overall protein sequence of a T cell receptor. This is defined as the identified V gene, the amino acid sequence of the CDR3 region and the identified J gene. Two rearrangements with the same bioidentity would be expected to demonstrate the same binding and activation behavior. Data Format: [Amino Acid]+[v_gene]+[j_gene]
TCR Nucleotide Sequence	String	The unique TCRB sequence identified as binding to the target.
Experiment	String	The experiment in which the binding was observed (joins to the subject-metadata.csv file).
ORF	String	The ORF in which this minigene target is located.
ORF Genebank ID	String	The identifier for the sequence from which the target was selected.
Amino Acid	String	The protein sequence of the minigene target.
Start Index in Genome	Integer	The 1-based index of the first base of the target within the genome.
End Index in Genome	Integer	The 1-based index of the last base of the target within the genome.

**Table 9:** Identity of TCRs bound per target in the peptide MIRA panel

Field	Value	Notes
ORF	String	The ORFs in which this target is located. Note some targets sit on multiple ORFs.
Amino Acids	String	The protein sequences that make up this target. Note some targets include multiple peptides.
Start Index in Genome	Integer	The 1-based index of the first base of leftmost peptide sequence within the genome.
End Index in Genome	Integer	The 1-based index of the last base of rightmost peptide sequence within the genome.
Hits	Integer	The unique count of TCRs identified as binding to the target, across all experiments.
TCR BioIdentity	String	Represents the overall protein sequence of a T cell receptor. This is defined as the identified V gene, the amino acid sequence of the CDR3 region and the identified J gene. Two rearrangements with the same bioidentity would be expected to demonstrate the same binding and activation behavior. Data Format: [Amino Acid]+[v_gene]+[j_gene]
TCR Nucleotide Sequence	String	The unique TCRB sequence identified as binding to the target.
Experiment	String	The experiment in which the binding was observed (joins to the subject-metadata.csv file).
ORF Coverage	String	The ORFs in which this target is located. Note some targets sit on multiple ORFs.
Amino Acids	String	The protein sequences that make up this target. Note some targets include multiple peptides.
Start Index in Genome	Integer	The 1-based index of the first base of leftmost peptide sequence within the genome.
End Index in Genome	Integer	The 1-based index of the last base of rightmost peptide sequence within the genome.
